# Quantum Mechanics Calculations, Basicity and Crystal Structure: The Route to Transition Metal Complexes of Azahelicenes

**DOI:** 10.3390/molecules17010463

**Published:** 2012-01-05

**Authors:** Tullio Caronna, Franca Castiglione, Antonino Famulari, Francesca Fontana, Luciana Malpezzi, Andrea Mele, Daniele Mendola, Isabella Natali Sora

**Affiliations:** 1 INSTM R.U. and Dipartimento di Ingegneria Industriale, Università di Bergamo, viale Marconi 5, 24044 Dalmine BG, Italy; 2 Dipartimento di Chimica, Materiali e Ingegneria Chimica G.Natta, Politecnico di Milano, via Mancinelli 7, 20123 Milano MI, Italy; 3 CNR-ICRM Istituto di Chimica del Riconoscimento Molecolare, via L. Mancinelli 7, 20131 Milano MI, Italy

**Keywords:** azahelicenes, DFT calculations, crystal structures, *N*-methylation, transition metal complexes

## Abstract

Quantum mechanics density functional calculations provided gas-phase electron distributions and proton affinities for several mono- and diaza[5]helicenes; computational results, together with experimental data concerning crystal structures and propensity to methylation of the nitrogen atom(s), provide a basis for designing azahelicene complexes with transition metal ions.

## 1. Introduction

Azahelicenes [1,2] constitute a class of heteroaromatic molecules whose properties represent promising features for several prospective applications in fields as diverse as optoelectronics [3], catalysis, sensors [4] and more. This is related on the one hand to their extended conjugated system, which imparts them large conductivity and polarizability, with high-order susceptibility term, and as a consequence a strong luminescence [5,6]. On the other hand, their intrinsic chirality due to helicity, with extremely high optical rotation, could be exploited both in the field of non-linear optics and for interactions with biomolecules [7,8]. Azahelicenes can aggregate into relatively stable supramolecular structures (π-stacking, polar interactions); in addition they exhibit a remarkably long triplet state lifetime [9].

**Figure 1:**
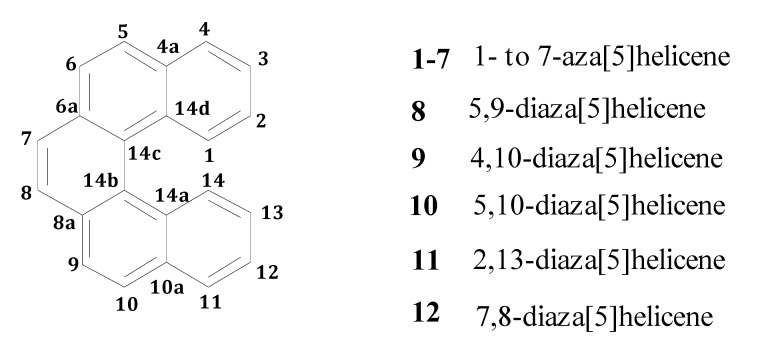
A generic [5]helicene with numbering of the skeletal positions.

This research group has a long-standing expertise in the synthesis and characterization of aza [5] helicenes (namely, helicenes containing one or more nitrogen atoms and constituted by five *ortho*-condensed aromatic rings, Figure 1) which led us, in recent years, to prepare all the monoaza [5] helicenes [9,10] and a considerable number of diaza [5] helicenes [9,11,12], following several synthetic strategies. Some crystallographic studies have been already carried out on these systems [9,13] in order to define the structural features mainly responsible for their supramolecular arrangement. We have now performed quantum mechanics density functional calculations concerning proton affinities and electronic distribution on several azahelicenes and some of their *N*-oxides, in order to provide a theoretical basis for the description of their behaviour. Computational results can be compared with experimental data concerning intermolecular distances and molecular packings obtained by x-ray diffraction, as well as with the observed reactivity of the nitrogen atoms towards *N*-alkylation. This collection of data may be of great help in designing possible transition metal complexes, which might prove useful for optoelectronic devices, due to the strong UV absorbance of helicenes and the possibility of energy transfer to the metal ion [14]. Two complexes with copper and platinum ions were also prepared and characterized.

## 2. Results and Discussion

The question of electron availability of the different atoms in the structures under investigation was addressed from different standpoints, with the ultimate purpose of providing a rationale for the design of new azahelicene-transition metal complexes.

Computational studies were carried out by means of density functional theory (DFT) for aza[5]helicenes in the gas-phase (see Figure 1 for a sketch of a generic helicene framework: One or two nitrogen atoms replace carbon atoms in the different positions indicated by the numbering), also including protonated systems, in order to estimate atomic charges and proton affinity (PA) of the nitrogen atoms.

Some mono- and diazahelicenes have been *N*-methylated and the findings concerning ease of formation of the methylazahelicenium salts fit well with calculated charge, hence confirming predictions of electron-donating ability by the nitrogen atoms.

Crystal structures of compound **12** and of its mono- and di-*N*-oxide (compounds **12a** and **12b**) were studied and compared, to find out structural features which might drive the formation of supramolecular aggregates, in view of exploiting the presence of oxygen atom(s) for metal ion complexation. Finally, metal complexes **5** and **12a** of azahelicenes with Pt(II) and Cu(II) were obtained and characterized.

### 2.1. DFT Calculations

All calculations were performed with the GAMESS-US suite of programs [15,16]. Stable low energy structures were obtained by DFT [17,18,19] calculations. In particular, the B3LYP hybrid functional [20,21,22] and the standard 6-311G** basis set [23] were adopted. In order to properly scan the potential energy surface (PES) of the systems under investigation, different starting guess geometries were considered, followed by full energy minimizations. A vibrational analysis was performed at the B3LYP/6-311G** level of the theory on optimised geometries, where a lack of imaginary frequencies confirmed that they represent minimum-energy structures.

**Figure 2:**
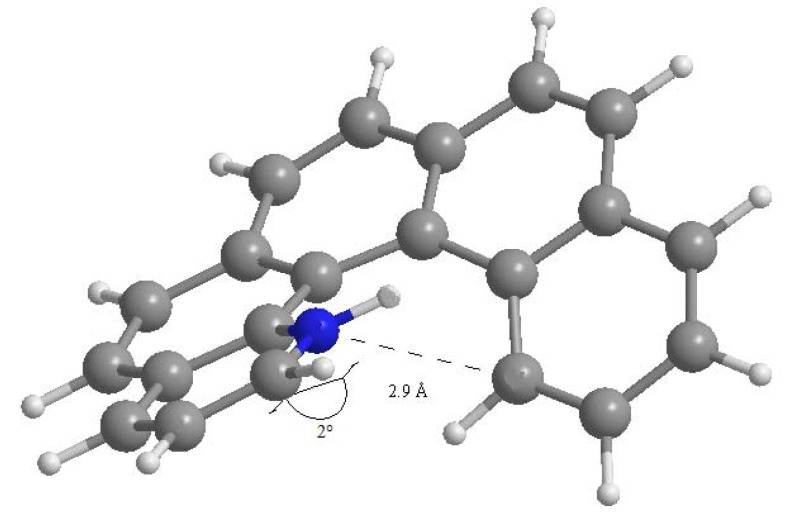
Computed structure of 1-aza[5]helicene with C14-N1 interatomic distance.

Atomic charges were calculated as the best fit to the Molecular Electrostatic Potential (MEP) obtained at the B3LYP/6-311G** level of the theory. Gavezzotti radii were used for charge fitting [24]. Gas phase PAs of molecules were estimated considering reaction (1):

*Helicene + H^+^ → Helicene-H^+^* (1)

and by means of Equation (2) [17]:

PA = −ΔE_el_ − ΔZPE + 5/2 RT (2)

where ΔE_el_ is the difference in electronic energy between the protonated and the unprotonated species, ΔZPE is the corresponding difference in zero point energies, R is the universal gas constant and T is the absolute temperature. The results concerning all possible monoaza[5]helicenes, together with quinoline as reference, are reported in Table 1. Compounds **1**–**7** correspond to monoaza [5] helicenes bearing a nitrogen atom in the positions 1 to 7 (see Figure 1). As can be seen, PAs are distributed in the range 995–1,010 kJ/mol; in the literature, compounds with PA around 1,000 kJ/mol are referred to as *superbases* [25,26]. Classical examples are the Hünig base (PA = 994 kJ/mol), diazabicycloundecene (PA = 1048 kJ/mol) or 1,8-bis(*N*,*N*-dimethylamino)naphthalene (PA = 1,028 kJ/mol) [27]. In comparison, the PA of quinoline is considerably lower. Protonation always reduces charge density on the nitrogen atom, but only in the case of **1** is the net charge on the nitrogen atom positive. This compound also shows a distinctly higher PA compared to all other terms of the series. Though not being of easy interpretation, this result might be related to the particular spatial arrangement of the protonated nitrogen with respect to the aromatic skeleton. In fact, the structure obtained upon *N*-protonation of **1** reveals a significant interaction between the proton and the fifth ring of the helicene backbone, which is reflected by the fact that the N–H bond is bent by about 2.0° towards the ring π system (Figure 2). Similar results have been reported in the case of 1-aza [6] helicene [28]. Further evidence of this interaction is the distance between C14 and N1 atoms, which was found to be 2.9 Å (less than the corresponding sum of van der Waals radii).

**Table 1:** Proton affinity values and estimated atomic charges on N atom for neutral and protonated monoaza[5]helicenescalculated at the B3LYP/6-311G** level of the theory (data pertaining to quinoline are used as reference).

Compound	Atomic charges on N atoms	PA (kJ/mol)
**1**	N1: −0.52	1,009.8
**1-H**^+^	N1: +0.06	
**2**	N2: −0.67	1,003.9
**2-H**^+^	N2: −0.21	
**3**	N3: −0.63	999.7
**3-H**^+^	N3: −0.06	
**4**	N4: −0.69	996.1
**4-H**^+^	N4: −0.28	
**5**	N5: −0.64	1,001.3
**5-H**^+^	N5: −0.26	
**6**	N6: −0.66	995.2
**6-H**^+^	N6: −0.21	
**7**	N7: −0.65	1,001.2
**7-H**^+^	N7: −0.20	
Quinoline	N: −0.61	970.9
Quinoline-H^+^	N: −0.09	

Several symmetric and asymmetric diaza[5]helicenes were also studied, namely the asymmetric 5,9-diazahelicene (**8**) and 4,10-diazahelicene (**9**) and the symmetric 5,10-diazahelicene (**10**) and 2,13-diazahelicene (**11**), as well as 7,8-diaza[5]helicene (**12**), for which both the mono- (**12a**) and di-*N*-oxide (**12b**) were also taken into account. PA was calculated for both nitrogen atoms, bearing to different values when the two atoms are non-equivalent. In the case of compounds **12a** and **12b**, PA was calculated for protonation of both the non-oxygenated nitrogen atom and the oxygen atom(s). For the sake of comparison, data pertaining 1,10-phenanthroline (**13**) are also reported (see Table 2).

**Table 2:** Proton affinity values and estimated atomic charges on electronegative atoms for neutral and protonated diaza[5]helicenes calculated at the B3LYP/6-311G** level of the theory. 1,10-phenanthroline (**13**) was included as reference.

Compound	Atomic charges on electronegative atoms	PA (kJ/mol) *
**8**	N5: −0.66 N9: −0.66	
**8-H**^+^(H5)	N5: −0.33 N9: −0.66	991.8
**8-H**^+^(H9)	N5: −0.58 N9: −0.27	976.3
**9**	N4: −0.69 N10: −0.64	
**9-H**^+^(H4)	N4: −0.27 N10: −0.59	979.1
**9-H**^+^(H10)	N4: −0.66 N10: −0.26	989.0
**10**	N5: −0.64 N10: −0.64	
**10-H**^+^(H5)	N5: −0.30 N10: −0.56	979.3
**10-H**^+^(H10)	N5: −0.56 N10: −0.30	979.2
**11**	N2: −0.65 N13: −0.65	
**11-H**^+^(H2)	N2: −0.16 N13: −0.65	997.2
**11-H**^+^(H13)	N2: −0.65 N13: −0.17	997.2
**12**	N7: −0.36 N8: −0.35	
**12-H**+(H7)	N7: +0.15 N8: −0.49	1,002.1
**12-H**+(H8)	N7: −0.49 N8: +0.15	1,002.4
**12a**	N7: +0.81 N8: −0.65 O7: −0.50	
**12a** -**H**+ (O7)	N7: +0.56 N8: −0.56 O7: −0.47	978.8
**12a** -**H**+ (N8)	N7: +0.56 N8: −0.38 O7: −0.37	957.1
**12b**	N7: +0.35 N8: +0.34	
	O7: −0.42 O8: −0.42	
**12b** -**H**+ (O7)	N7: +0.12 N8: +0.39	973.0
	O7: −0.39 O8: −0.41	
**12b** -**H**+ (O8)	N7: +0.40 N8: +0.11	973.1
	O7: −0.41 O8: −0.38	
**13**	N1: +0.05 N9: −0.55	1,015.5
**13-H**^+^	N1: −0.56 N9: +0.06	1,015.6

* PA values pertain to neutral fragments. Due to the presence of different protonation sites, for clarity PAs are here referred to corresponding protonated species.

It is to be noted that in diazahelicenes where the two nitrogen atoms are located in different rings, *i.e.*, molecules **8**, **9**, **10** and **11**, the electronegative atoms bear a larger negative charge than do the nitrogen atoms in diazahelicene **12**, where the nitrogen atoms are adjacent, in a structure somewhat resembling an azo compound. For compound **12** it is evident that the atomic charges on adjacent nitrogen atoms don’t reflect their PA; this is actually a more complex property and depends on the general arrangement of the molecule. In the mono-*N*-oxide **12a** the PA is higher for the protonated oxygen than for the protonated nitrogen in position 8. This latter nitrogen atom’s electron availability is likely affected by the inductive effect exerted by the nearby oxygen-bonded nitrogen 7, which bears a partial positive charge. Besides, this observation can be related to crystallographic data (*vide infra*) concerning the oxygen atom distribution. In the unsymmetrical compound **8** it can be observed that the PA is stronger for the nitrogen atom in position 5 than for the one in position 9. Besides, in this case protonation on nitrogen in position 5 determines hardly any effect on the other (non-conjugated) nitrogen, whose net charge is essentially the same as in the unprotonated molecule; when nitrogen in position 9 is protonated, on the other hand, an effect is present on the other nitrogen, which is even more depleted of negative charge than in the case of symmetrical, conjugated compound **10**.

These calculations give an idea of the availability of lone pairs for a dative bond in a metal-helicene coordination complexes, but the formation and the thermodynamic stability of these latter are also influenced by other factors, such as steric hindrance, ionic radius of the metallic ion and the chelating effect, that can severely affect the stability of one metal complex compared to another.

### 2.2. *N*-Methylation of Mono-and Diazahelicenes

Quaternary *N*-alkylazahelicenium salts are usually easily obtained by direct treatment of the parent azahelicene with an alkylating agent such as an alkyl iodide or dimethyl sulfate. All monoaza[5]helicenes can be alkylated this way, with the possible exception of **1**, whose steric requirements are higher. The nitrogen atom acquires a positive net charge and the carbon atoms on the molecular framework are depleted of electron density, as can be seen from the comparison of chemical shifts in the ^1^H-NMR spectra taken for monoazahelicene **5** and its *N*-methylated quaternary salt **5a**; especially the position adjacent to the quaternary nitrogen (in this case, position 6) is severely deshielded, with a chemical shift of 11.65 ppm for **5a** comparing to a value of 9.41 ppm for **5**. Full characterization of *N*-methyl-5-aza[5]helicenium iodide, taken as a representative example of this series of compounds, is given in the Supplementary Material.

**Figure 3:**
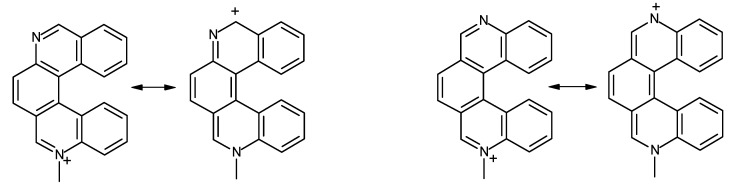
Limit structures of monomethylated diazahelicenes **8** and **10** showing delocalization of positive charge onto the ring bearing the unprotonated nitrogen atom.

As already mentioned, the data in Table 2 clearly show that the nitrogen atoms in diazahelicene **12** bear a definitely lower negative charge than do nitrogen atoms in other azahelicenes; this result is reflected in the behaviour of compound **12**, which does not give a quaternary salt in the conditions in which all other studied compounds do. Moreover, charge distribution data concerning diazahelicenes other than **12** are confirmed by experimental findings: in facts, for neutral compounds **8**, **9**, **10** and **11** the electron density is about the same for the two nitrogens, but when one of them is protonated, the other one is strongly depleted of electrons, except in the case of compound **8**. This could be explained by the fact that in the structures **9**–**11** the two nitrogen atoms are conjugated (Figure 3, right), while in compound **8** (Figure 3, left) they are not.

**Figure 4:**
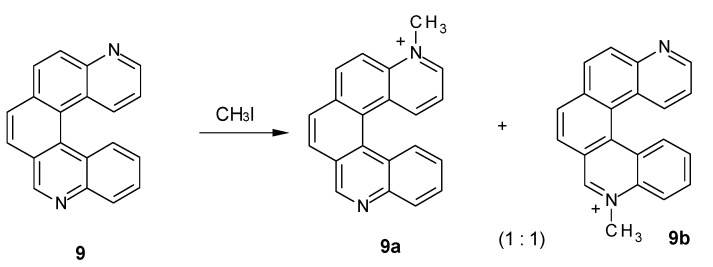
*N*-Methylation of diazahelicene (**9**)*.*

Consistently, when performing methylation of compound **9**, whose nitrogen atoms are unsymmetrical but conjugated, only one of them is methylated. Both monomethylated products were observed (Figure 4), while no dimethylated derivative was detected. Indeed, the ESI mass spectrum showed an intense peak at *m/z* 295, assigned to a monomethylated species [C_21_H_15_N_2_]^+^, but no peak at *m/z* 155, corresponding to the doubly charged dimethylated specie [C_22_H_18_N_2_]^2+^ was detected. The NMR spectrum of the reaction mixture (see Supplementary Material) showed 24 resolved multiplets, consistent with the presence of two regioisomers in solution. The signals corresponding to the protons most sensitive to *N*-methylation, H9, H1, H2 and H3, showed homologous counterparts at lower fields, confirming the presence of both **9a** and **9b** (e.g., the singlets at 10.20 and 9.57 ppm can be assigned to H9 of **9b** and H9 of **9a**). Integration of the above mentioned peaks indicated a 1:1 ratio between the two regioisomers.

On the other hand, when methylation was performed on unsymmetrical nonconjugated diazahelicene **8**, the ESI-MS spectrum of the crude reaction mixture showed, besides the peak at *m/z* 295, also an intense peak at *m/z* 155, indicating the presence of a doubly charged species of molar mass 310, corresponding to the dimethylated product (C_22_H_18_N_2_)^2+^.

A consequence of this is that by exploiting this effect, it should be possible to *N*-alkylate one of the nitrogens, thus making the helicene much more water-soluble, while keeping the other nitrogen available for complexation, which might prove useful for biological studies [7,8].

### 2.3. Crystal Structures and Crystal Packing

Several mono- and diaza [5] helicenes have been already studied by single-crystal X-ray diffraction [9,13] ; in this work we discuss the x-ray diffraction analysis of compounds **12**, **12a** and **12b**. The molecular geometry of these helicenes shows the usual alternation of short and long C–C bonds.

The three molecules **12**, **12a** and **12b** have a helical shape with a true (as in symmetric **12** and **12b**) or pseudo (as in asymmetric **12a**) twofold axis, which bisects the N7–N8 and the C14b–C14c bonds.

In **12a** the O atom was found to be disordered over two positions, being partly bonded to N7 and partly to N8, with site occupation factors of 0.68 and 0.32 respectively. The N7-N8 bond distances are similar for **12** and **12a** [1.308 (3) Å] and slightly longer for **12b** [1.355(3) Å]. The torsion of the whole molecule about the central ring, typically evaluated by the torsion angle around C14b and C14c bond, shows values of 23.9(3), 25.9(3) and 25.8(4)° for **12**, **12a** and **12b** in sequence. The molecular volumes increase with increasing oxygen content with values 1365.9(3), 1401.2(2) and 1456.9(4) Å^3^, respectively, for **12**, **12a** and **12b**.

**Figure 5:**
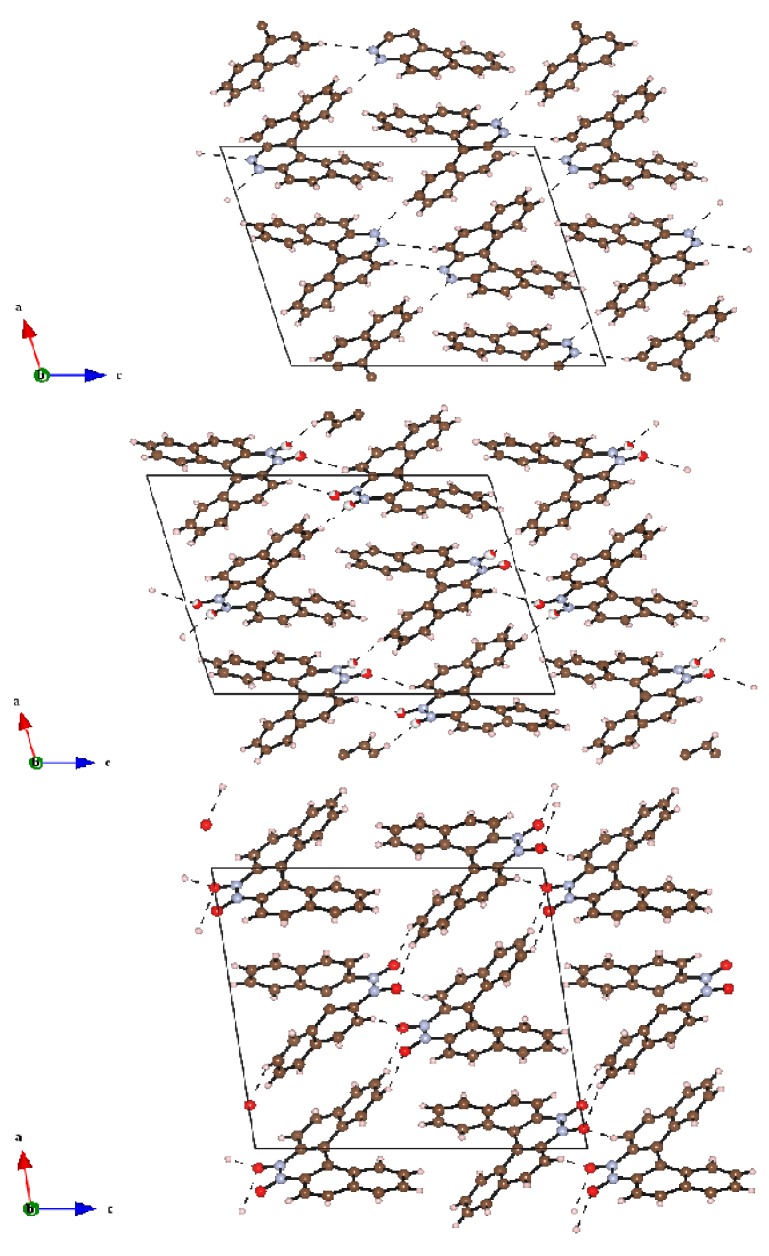
Projections along the b axis of (**a**) **12**, (**b**) **12a** and (**c**) **12b**. For compound **12a**, the oxygen atoms are found disordered over two positions with occupation factors less than unity: these atoms are displayed as circle graphs.

It is interesting to note that the compounds crystallize readily and easily, sometimes directly in the course of the chromatographic separation upon slow evaporation of the solvent from the collected chromatographic fractions, while for most other azahelicenes obtaining satisfactory single crystals is usually much less straightforward. This might point out that crystal packing energy is particularly strong for these molecules, driving the formation of well-ordered supramolecular structures. As it can be observed in Figure 5, which shows the crystal packing of **12**, **12a** and **12b**, the structures of the *N*-oxides are definitely more ordered compared to the structures described in the literature for other diazahelicenes [9,13].

For compound **12**, hydrogen bonding is modest due to its lack of good donor groups and short interactions are limited to C-H---N or C-H---O type (see Table 3). In **12a**, where the oxygen atom is affected by disorder, the shortest possible C-H---O contact occurs at 2.871(5) Å, while in **12b** the shortest C-H---O contact occurs at 3.329(3)Å.

**Table 3:** Relevant intermolecular distances.

Compound	C-H---X contacts	C----X distances	C-H---X angles
**12**	C(2)-H(2)---N(8)	3.452(3) Å	155.3(2)°
	C(6)-H(6)---N(7)	3.808(3) Å	156.0(2)°
**12a**	C(2)-H(2)---O(16)	2.871(5) Å	137.8(2)°
	C(6)-H(6)---O(15)	3.329(3) Å	143.6(2)°
**12b**	C(2)-H(2)--- O(16)	3.399(3) Å	147.4(2)°
	C(3)-H(3)--- O(15)	3.374(3) Å	159.5(2)°
	C(6)-H(6)--- O(15)	3.329(3) Å	153.4(2)°

At a supramolecular level anti-parallel stacking between the central aromatic rings is observed in the three helicenes. The structure consists of homochiral columns parallel to the *b* axis and columns of alternate *R*- and *S*-molecules perpendicular to the *ac* plane. The packing coefficient, evaluated as the ratio between *V_mol_* (the volume occupied by van der Waals atomic spheres for the gas phase molecule) and *V/Z* (where *V* is the unit cell volume and *Z* is the number of molecules in the unit cell) gives information about the packing efficiency. Going from **12** to **12b**, the packing coefficients are 0.769, 0.776 and 0.793, and may be correlated with the number of short intermolecular interactions.

### 2.4. Transition Metal Complexes

Transition metal complex formation was attempted starting both from plain azahelicenes and from the N-oxides; we discuss here the characterization of the complexes formed by 5-aza[5]helicene **5** with PtCl_2_(PPh_3_)_2_ (**14**) and by 7,8-diaza[5]helicene-*N*-oxide **12a** with CuCl_2_ (**15**). Due to the failure to obtain single crystals of appropriate shape and size for x-ray diffraction, characterization was based on NMR and mass spectrometry, as detailed below.

**Figure 6:**
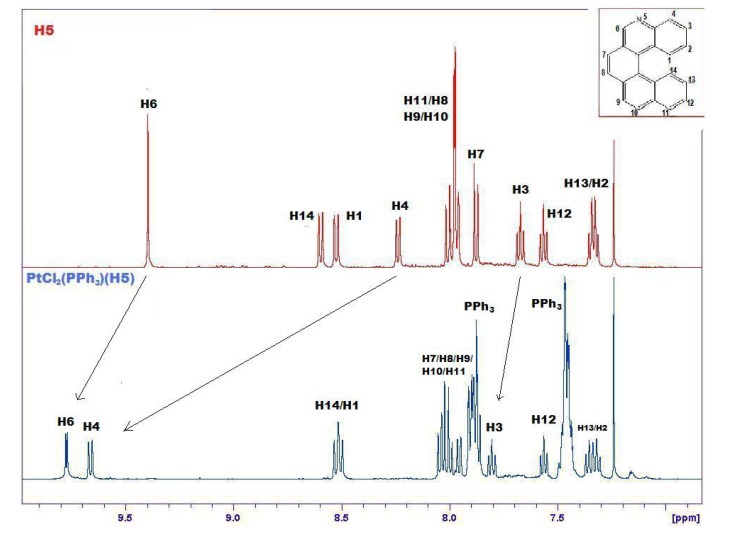
Comparison between ^1^H-NMR spectra of free **5** (upper, red) and its complex **14** with PtCl_2_(PPh_3_) (lower, blue) in the range 7–10 ppm. The most significant shifts are evidenced with an arrow. Signals related to PPh_3_ are labeled.

The formation of **14** is demonstrated by many pieces of evidence. Figure 6 compares the ^1^H-NMR spectra of **5** and **14**, evidencing marked downfield shifts due to complexation. Only the relevant range of chemical shifts, namely the range comprising aromatic hydrogens between 7 and 10 ppm, is shown in Figure 5. From this spectrum it is possible to observe selective shifts of signals belonging to H atoms located in the proximity of the Pt complexation site. Attribution of signals to specific protons of the complex was confirmed by selective decoupling. The most significant shifts are evidenced with an arrow and concern mainly atoms close to the nitrogen atom of helicene.

**Figure 7:**
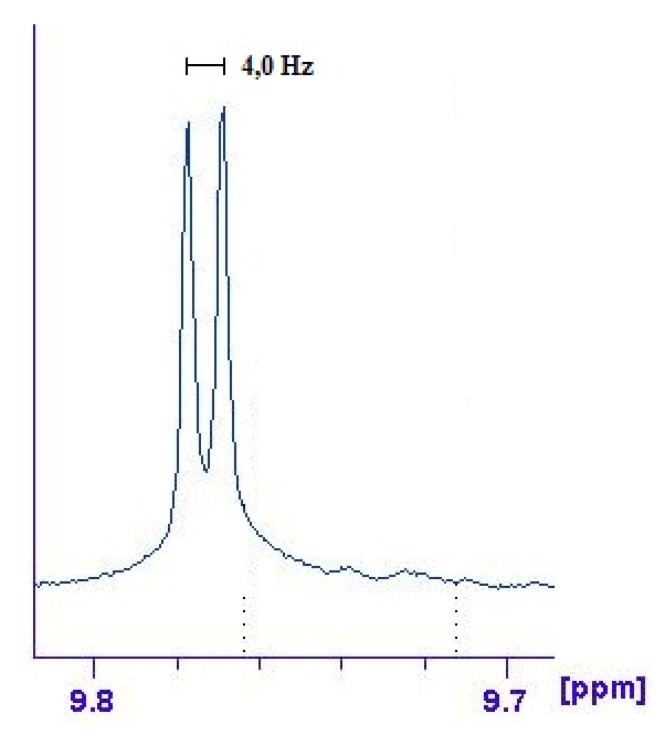
Expanded section of the ^1^H-NMR spectrum of complex **14**, focused onthe areaassignedtoproton6 of 5-aza[5]helicene molecule. A coupling constant of 4 Hz, absent in the spectrum of the free molecule, can be observed.

The signal attributed to H6 of helicene (see Figure 1) shows a coupling constant of about 4 Hz (enlarged in Figure 7), absent in the upper spectrum, which can be ascribed to a *^3^J*_Pt-H_ constant [29]. ^31^P- and ^195^Pt-NMR spectra show a significative variation in their chemical shifts and coupling constants compared to the precursor (see Experimental and Supplementary Material).

**Figure 8:**
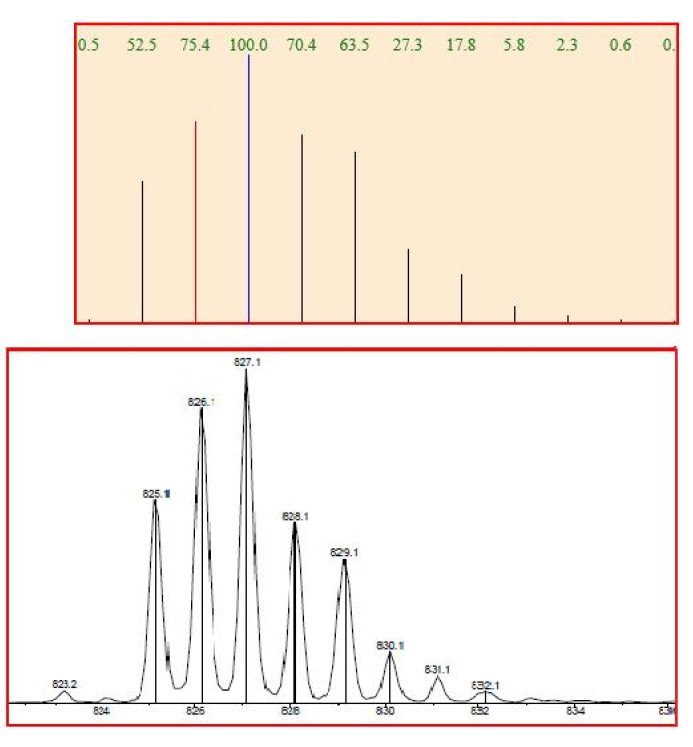
Calculated (upper) and experimental (lower) ESI-MS spectra for compound **14**.

ESI-MS spectrum confirmed the presence of a Pt complex with azahelicene. The complex was introduced in the ESI source by direct infusion of a CH_3_CN solution. Ligand exchange occurred in the ESI source giving rise to the cationized specie [PtCl **5** P(Ph)_3_ CH_3_CN]^+^ of molecular formula [C_41_H_31_N_2_PClPt]^+^. Figure 8 shows an excellent match between the calculated and the experimental isotopic cluster for such platinum/azahelicene complex.

**Figure 9:**
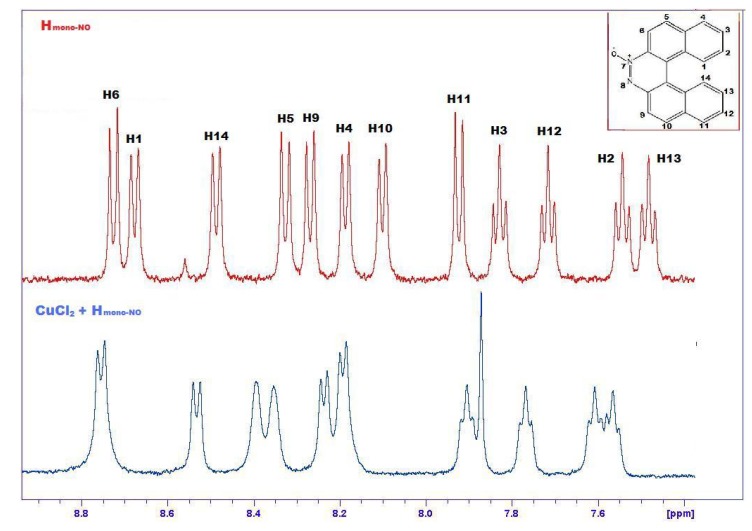
Comparison between ^1^H-NMR spectra of **12a** (upper, red) and **15** (lower, blue).

For complex **15** the ^1^H-NMR spectrum shows a less marked shift of the hydrogen signals and a significant enlargement of the lines, due to the paramagnetic effect of Cu (Figure 9) [30,31].

**Figure 10:**
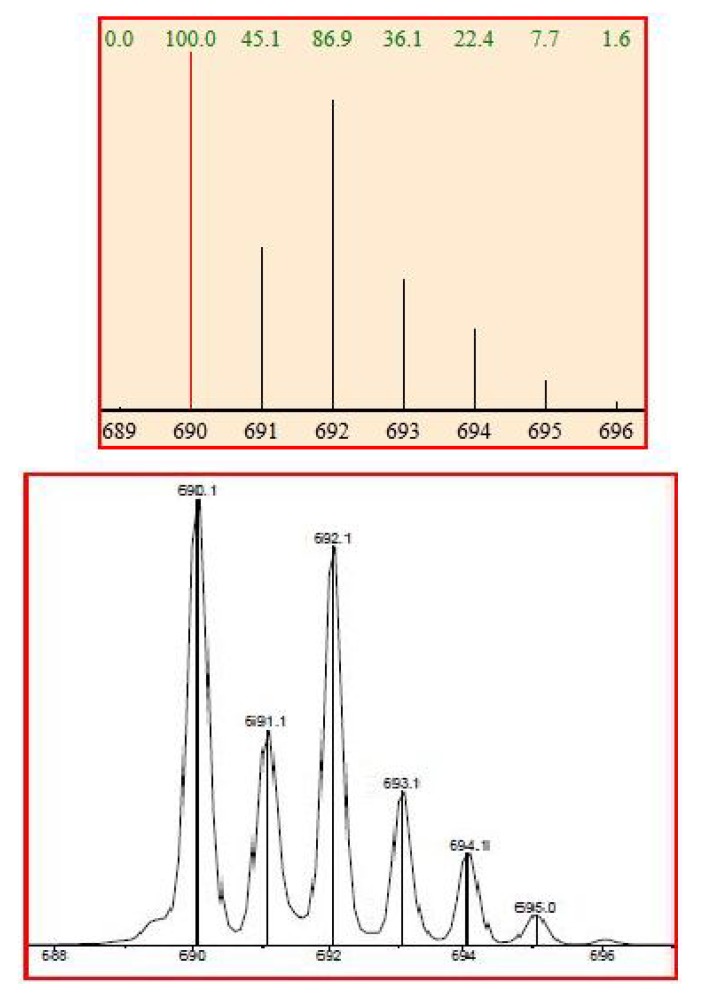
Comparison between calculated (upper) and experimental ESI-MS spectra for **15** (lower).

The corresponding ESI-MS spectrum shows an excellent correspondence between calculated and experimental data (Figure 10) and shows the formation of a complex Cu/**15** in ratio 1:2, corresponding to the cationized species [C_40_H_24_N_4_O_2_CuCl]^+^.

## 3. Experimental

### 3.1. General

Azahelicenes **1**–**7** were synthesized as detailed in references [9,10]. The preparation of diazahelicenes **8**, **9** and **10** is also reported in [9], while compound **11** is described in reference [11] and compounds **12**, **12a** and **12b** were prepared as described in reference [12].

All other reagents and solvents were bought from Sigma-Aldrich and used either as such or after opportune drying where required. All complexation reactions were conducted under nitrogen using Schlenk techniques. ^1^H-NMR spectra were recorded on a Bruker Avance 500 spectrometer equipped with a QNP switchable probe ^13^C-^31^P-^19^F-^1^H and operating at proton resonance frequency of 500 MHz. The products were dissolved in CDCl_3_ or CD_3_OD and referenced to Si(CH_3_)_4_ for ^1^H and ^13^C. Multinuclear NMR spectra were performed on a Bruker DRX 500 spectrometer, equipped with a BBI broadband probe. For ^31^P-NMR spectra all samples were equipped with a coaxial capillary filled with a solution of 85% of H_3_PO_4_ in D_2_O, used as zero reference; ^195^Pt-NMR spectra were referenced to a solution of H_2_PtCl_6_ in D_2_O. The crystal structures of compounds **12**, **12a** and **12b** have been deposited at the Cambridge Crystallographic Data Centre with deposition numbers CCDC 859770, CCDC 859771 and CCDC 859772. These data can be obtained free of charge via http://www.ccdc.cam.ac.uk/conts/retrieving.html.

### 3.2. Synthesis of Quaternary N-Methylazahelicenium Salts

The azahelicene (0.1 mmol) was dissolved in CCl_4_ (5 mL) and a large excess (5 mmol) of methyl iodide was added; the mixture was refluxed overnight with stirring, wherupon a yellow precipitate formed that was collected by filtration. Yields exceed 90%. The *N*-methyl-5-aza[5]helicenium iodide **5a** obtained in this way from **5** has a melting point of 238–240 °C; full characterization for this compound including IR, UV-Vis, ^1^H-NMR, ^13^C-NMR, ESI-mass spectrum and single crystal X-ray diffraction data is provided in the Supplementary Material.

### 3.3. Synthesis and Characterization of Metal Complexes

The preparation of *cis/trans*-PtCl_2_(NCEt)_2_ was carried out by a literature method [32], starting from commercial K_2_PtCl_4_. The terms s, d, t, q, m indicate respectively singlet, doublet, triplet, quartet, multiplet; b is for broad and, dt means double triplet. Positive-ion ESI-MS was performed on Esquire 3000 plus ion-trap mass spectrometer (Bruker Daltonik, Bremen, Germany) equipped with an ESI source. Sample solutions were introduced into the ion source at a flow-rate 4 μL m^−1^, capillary voltage 3.8 kV, drying gas temperature 250 °C, drying gas flow rate 5 L m^−1^, nebulizer pressure 14 psi. Nitrogen was used as both nebulizing gas and drying gas.

#### 3.3.1. cis-PtCl_2_(NCEt)(PPh_3_) * [33]*

PtCl_2_(NCEt)_2_ (0.540 g, 1.44 mmol), triphenylphosphine (0.370 g, 1.41 mmol) and propionitrile (5.0 mL) were placed in a 50 mL Carius tube equipped with a magnetic stirrer, then the tube was sealed under an inert atmosphere. The mixture was heated under stirring during 12 h at 145 °C to give a clear yellow solution. Heating was stopped and the solution was allowed to cool down slowly. The resulting pale yellow crystalline solid was filtered off under nitrogen and the solvent removed under vacuum to constant weight to afford 0.563 g of product (0.965 mmol, 67% yield). ^1^H-NMR (CDCl_3_): 7.78–7.45 (15H, phosphinic hydrogens); 2.14 (q, *^2^J*_H-H_ = 7.5 Hz, 2H, CH_2_); 0.87 (t, *^2^J*_H-H_ = 7.5 Hz, 3H, CH_3_); ^13^C-NMR (CDCl_3_): 134.6 (d, *J*_C-P_ = 9.7 Hz), 131.5 (s), 128.4 (d, *J*_C-P_ = 10.6 Hz) e 126.3 (d, *J*_C-P_ = 66.5 Hz) aromatics; 118.0 (s, NC); 12.3 (s, CH_2_); 8.8 (s, CH_3_); ^31^P-NMR (CDCl_3_): 5.2, *^1^J*_P-Pt_ = 3560 Hz; ^195^Pt-NMR (CDCl_3_): −3595 (d), *^1^J*_Pt-P_ = 3560 Hz.

#### 3.3.2. PtCl_2_(PPh_3_)*(**5**)*

A 50 mL Schlenk tube was loaded with PtCl_2_(NCEt)(PPh_3_) (50.0 mg, 0.086 mmol), **5** (29 mg, 0.104 mmol) and propionitrile (8.0 mL). The mixture was heated under stirring at the solvent reflux temperature (97 °C) for 6 h, then the clear yellow solution was evaporated to give a brown amorphous powder that was recrystallized from 1,2-dichloroethane with the addition of *n*-heptane to afford 34.6 mg of product (42.9% yield). ^1^H-NMR (CDCl_3_): 9.77 (d, *^3^J*_Pt-H_ = 4.0 Hz, 1H, H6); 9.66 (d, *^2^J*_H-H_ = 8.5 Hz, 1H, H4); 8.52 (t, *^2^J*_H-H_ = 9.6 Hz, 2H, H1/H14); 8.05–7.95 (m, 5H, H7/H8/H9/H10/H11); 7.91–7.86 (m, 8H, PPh_3_); 7.80 (t, *^2^J*_H-H_ = 7.1 Hz, 1H, H3); 7.56 (t, *^2^J*_H-H_ =7.4 Hz, 1H, H12); 7.50–7.43 (m, 8H, PPh_3_); 7.37–7.30 (dt, *^2^J*_H-H_ = 8.3 Hz, 2H, H2/H13); ^31^P-NMR (CDCl_3_): 3.1, *^1^J*_P-Pt_ = 3660 Hz; ^195^Pt-NMR (CDCl_3_): −3522 (d), *^1^J*_Pt-P_ = 3660 Hz.

#### 3.3.3. CuCl*(**12a**)_2_*

A solution of CuCl_2_ (100.0 mg, 0.744 mmol) in CH_3_OH (3 mL) was added to a solution of **12a** (28.0 mg, 0.094 mmol) in 1,2-dichloroethane (2 mL). The resulting green mixture was heated under stirring at the solvent reflux temperature (approximately 80 °C) for 4 h. Then the solvent was evaporated under vacuum and the resulting solid powder was treated with CH_2_Cl_2_, to dissolve only the helicene complex. The resulting CH_2_Cl_2_ solution was evaporated under vacuum to give 24.0 mg of product (a yield of 70.2%). ^1^H-NMR (CD_3_OD): 8.75 (d, *^3^J*_Pt-H_ = 6.5 Hz, 2H, H1/H6); 8.53 (d, *^2^J*_H-H_ = 8.4 Hz, 1H, H14); 8.39 (s, b, 1H, H5); 8.35 (s, b, 1H, H9); 8.24 (d, *^2^J*_H-H_ = 8.2, 1 H, H4); 8.19 (d, *^2^J*_H-H_ = 8.1 Hz, 2H, H10/H11); 7.90 (t, *^2^J*_H-H_ =6.9 Hz, 1H, H3); 7.77 (t, *^2^J*_H-H_ = 6.8 Hz, 1H, H12); 7.61 (t, *^2^J*_H-H_ = 7.3 Hz, 1H, H2); 7.57 (t, *^2^J*_H-H_ = 7.3 Hz, 1H, H2).

## 4. Conclusions

Quantum mechanics DFT calculations were performed on a series of mono-and diaza[5]helicenes and some of their N-oxides; values of atomic charge, as well as proton affinities, were obtained for the electronegative atoms. The findings put into evidence that most of these compounds show a considerable electron density on the nitrogen atom(s), which in principle should indicate the viability of the preparation of complexes with metal ions. The same calculations were also performed on the protonated forms of the same molecules, in which the electron density is modified depending on which of the available electronegative atom(s) is protonated. The calculated data show that protonation on one nitrogen of a diazahelicene depletes the other nitrogen of electron density only in the cases where the two nitrogens are conjugated; in cases where they are not, one of the nitrogen atoms could be quaternarized with the purpose of increasing water solubility of the helicene, while the other one would remain free for metal ion complexation. N-methylation experiments are reported, which support the findings of the quantum mechanics DFT calculations for diazahelicenes. A Pt complex of a monoaza[5]helicene is obtained and characterized.

Single crystal structures of 7,8-diaza[5]helicene and of its mono- and di-N-oxides were resolved and compared with the structures of other diazahelicenes reported in the literature, evidencing the presence of strong intermolecular interactions, driving the formation of well-ordered structures in the solid state. While in other cases interactions like π-stacking of the aromatic rings are mainly responsible for the spatial arrangement of the molecules, the presently discussed structures crystallize very easily in structures driven by the formation of Van der Waals attractions and hydrogen bonding. This suggested the possibility of metal ion complex formation despite the relatively low charge located on the nitrogen atoms, by exploiting the presence of the negatively charged oxygen: a Cu(II) complex was actually prepared from 7,8-diaza[5]helicene mono-N-oxide. Work is in progress towards the preparation of complexes with other metal ions and using different azahelicenes, for a range of prospective applications.

## Supplementary Materials

Supplementary materials can be accessed at: http://www.mdpi.com/1420-3049/17/1/463/s1.
