# Supplementary material for: Quantum Mechanics Calculations, Basicity and Crystal Structure: The Route to Transition Metal Complexes of Azahelicenes
Source: Molecules. 2012 Jan 5;17(1):463–79. doi: 10.3390/molecules17010463 (PMC6268832; doi:10.3390/molecules17010463)

*Supporting Information*

Quantum Mechanics Calculations, Basicity and Crystal Structure: The Route to Transition Metal Complexes with Azahelicenes

Tullio Caronna ^1^, Franca Castiglione ^2^, Antonino Famulari ^2^, Francesca Fontana ^1,2^,*,
Luciana Malpezzi ^2^, Andrea Mele ^2,3^, Daniele Mendola^2^ and Isabella Natali Sora ^1,2^

^1^ INSTM R.U. and Dipartimento di Ingegneria Industriale, Università di Bergamo, viale Marconi 5, 24044 Dalmine BG, Italy

^2^ Dipartimento di Chimica, Materiali e Ingegneria Chimica G.Natta, Politecnico di Milano,
via Mancinelli 7, 20123 Milano MI, Italy

^3^ CNR-ICRM Istituto di Chimica del Riconoscimento Molecolare, via L. Mancinelli 7,
20131 Milano MI, Italy

***** Author to whom correspondence should be addressed; E-Mail: francesca.fontana@unibg.it;
Tel.: +39-035-205-2322; Fax: +39-035-205-2077.

**1. Characterization of *N*-Methyl-5-aza[5]helicenium iodide (5a)**

*1.1. General Procedures*

^1^H-NMR spectra were recorded on a Bruker Avance 500 spectrometer operating at proton resonance frequency of 500 MHz. The products were dissolved either in CDCl_3_ or CD_3_OD with TMS (tetramethylsilane) as internal reference. ^13^C-NMR spectra were acquired under proton broad-band decoupling (WALTZ16 decoupling sequence). The full assignment of all non-quaternary C atoms was achieved by two-dimensional C-H correlation spectroscopy via one-bond coupling (HSQC). The assignment of quaternary carbons was supported by multiple-bond C-H correlation spectroscopy (HMBC). Electron ionization mass spectra were recorded on a Finnigan MAT TSQ 70 instrument; the samples were introduced in the spectrometer source by direct probe insertion. The UV-visible absorption spectra were recorded on a Thermo Nicolet Evolution UV-Vis-500 spectrophotometer, Vision 32 software, in a 10^−4^ M methanol solution, using a 1 cm pathlength cell. The FT-IR spectra were recorded in solid phase on a Thermo Avatar 370 equipped with an Attenuated Total Reflectance (ATR) accessory. All the spectra were recorded at room temperature.

*1.2. Data*

Elemental analysis calculated (%) for C_22_H_16_NI C 62.72, H 3.83, N 3.33; Exp. (%) C 62.93, H 3.84, N 3.32. Melting point 238–240 °C, compared to a melting point of 198–200 °C for compound **5**. The ESI-MS spectra of the *N*-methyl derivative acquired in the positive ion polarity show, as expected, a very strong signal at *m/z* 294 (M^+^), whilst the spectrum in the negative ion polarity confirms the presence of the negative I^−^ counterion.

*1.3. NMR Spectra*

**Table S1.** ^1^H-NMR spectra of **5** and of **5a** in different solvents.

|  | (**5**), CDCl_3_ *δ (ppm) J*(Hz) | (**5a**) CDCl_3_ *δ (ppm) J*(Hz) | (**5a**) CD_3_OD *δ (ppm) J*(Hz) |
| --- | --- | --- | --- |
| H1 | *8.54,* dd 1.4; 8.7 | *8.79,* dd 1.4; 8.5 | *8.77,* dd 1.5; 8.6 |
| H2 | *7.35,* ddd 1.4; 6.9; 8.7 | *7.65,* ddd 1.2; 7.1; 8.5 | *7.75-7.72* m |
| H3 | *7.69,* ddd 1.4; 6.9; 8.3 | *8.01,* ddd 1.4; 7.1; 8.7 | *8.11,* ddd 1.5; 7.1; 8.7 |
| H4 | *8.25* dd 1.4; 8.3 | *8.31* dd 1.2; 8.7 | *8.57* dd 1.2; 8.7 |
| H6 | *9.41* s | *11.65* s | *10.14* s |
| H7 | *7.89* d 8.7[a] | *8.78* d 8.3 | *8.40* d 8.3 |
| H8 | *8.03* d 8.7[a] | *8.22* d 8.3 | *8.38* d 8.3 |
| H9 | [b] | *7.98* d 8.7 | *7.98* d 8.6 |
| H10 | [b] | *8.24* d 8.7 | *8.37* d 8.6 |
| H11 | *7.98* dd 1.4; 8.3 | *8.07* dd 1.4; 8.3 | *8.17* dd 1.5; 8.1 |
| H12 | *7.58,* ddd 1.4; 6.9; 8.3 | *7.69,* ddd 1.2; 7.0; 8.3 | *7.75–7.72* m |
| H13 | *7.36,* ddd 1.4; 6.9; 8.3 | *7.44,* ddd 1.4; 7.0; 8.5 | *7.47,* ddd 1.5; 7.0; 8.5 |
| H14 | *8.62* br d 8.3 | *8.40* br d 8.5 | *8.40* br d 8.5 |
| CH_3_ | *--* | *5.02* s | *5.02* s |

[a]: assignments can be reversed; [b]: 7.97–8.01, multiplet.

**Table S2.** ^13^C-NMR spectrum of **5a**.

| **carbon atom** | **frequency** | **carbon atom** | **frequency** |
| --- | --- | --- | --- |
| C1 | 130.48 | C13 | 127.62 |
| C2 | 129.36 | C14 | 128.88 |
| C3 | 133.73 | C8a | 140.12 |
| C4 | 119.85 | C14d | 135.91 |
| C6 | 154.45 | C14a | 134.83 |
| C7 | 132.52 | C10a | 131.37 |
| C8 | 132.52 | C4a | 127.51 |
| C9 | 127.11 | C14b | 125.59 |
| C10 | 134.88 | C6a, C14c | 135.91, 126.10 |
| C11 | 129.88 | CH_3_ | 46.15 |
| C12 | 129.62 |  |  |

**Figure S1.** ^1^H-NMR spectrum of **5a**.


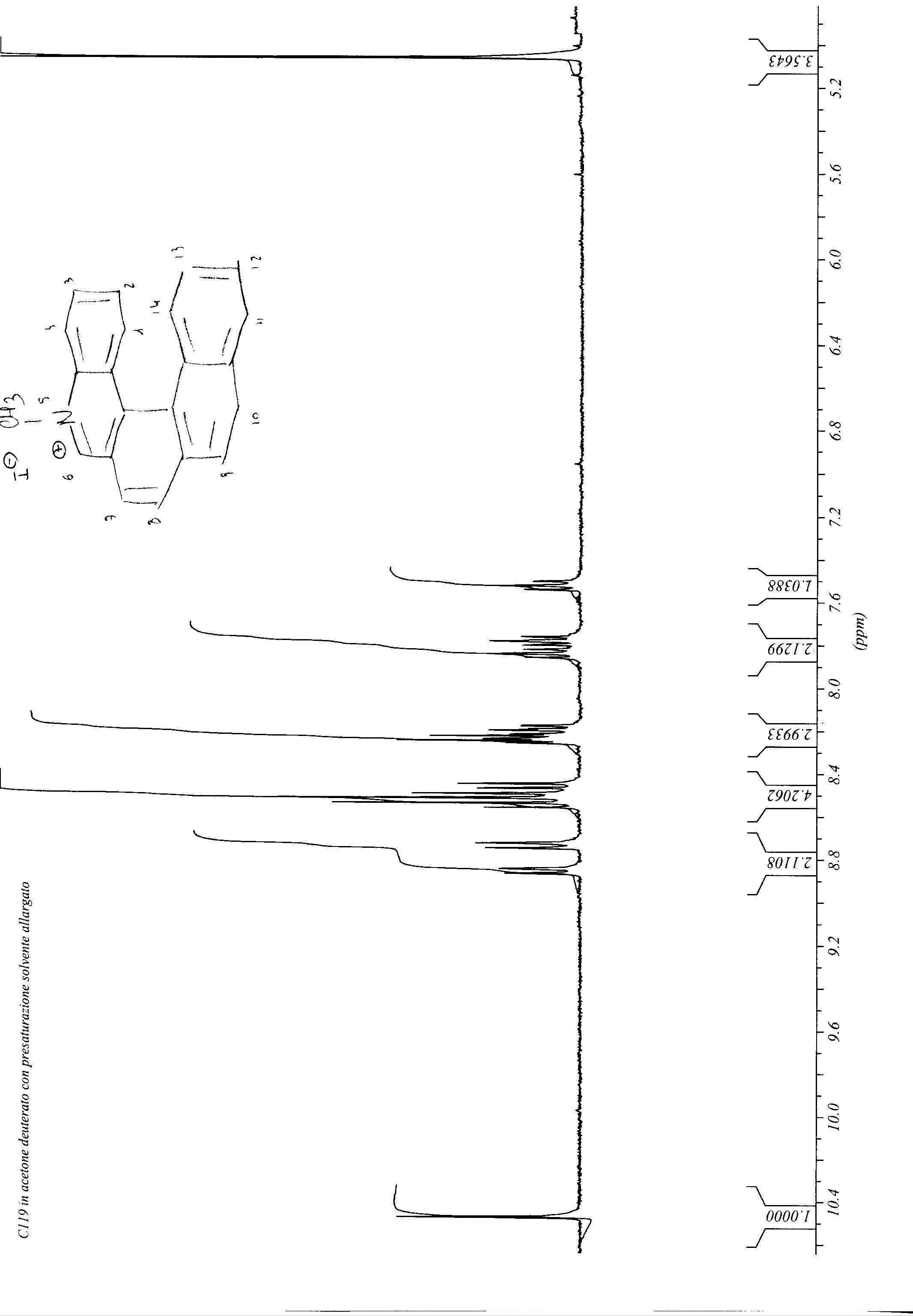


**Figure S1b.** ^1^H-NMR spectrum of **5a** (~7.4–8.8 ppm).


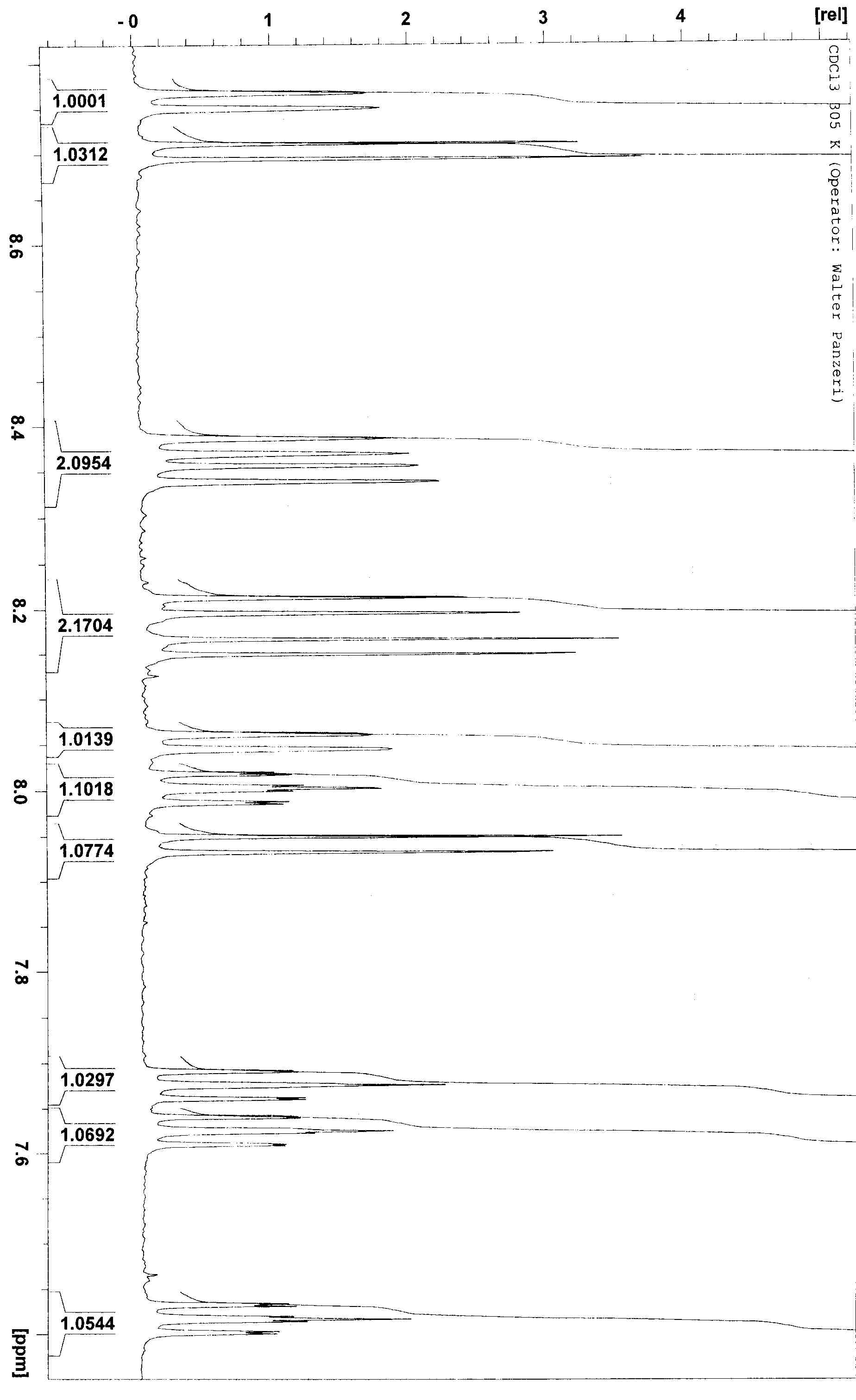


*1.4. IR and UV Spectroscopy*

The bands of medium intensity at 1620, 1592, 1527, 1370, 848 and 758 nm are present in the spectrum of (**5a**) while they are absent in (**5**), which shows instead weaker signals at 1580, 1494, 1433, 1380, 1218 and strong bands at 836, 768, 750 nm, absent in the spectra of (**5a**). The signal at 1370 nm in the quaternary salts can be attributed to the methyl group.

**Figure S2.** IR spectra of **5** (above) and **5a** (below).


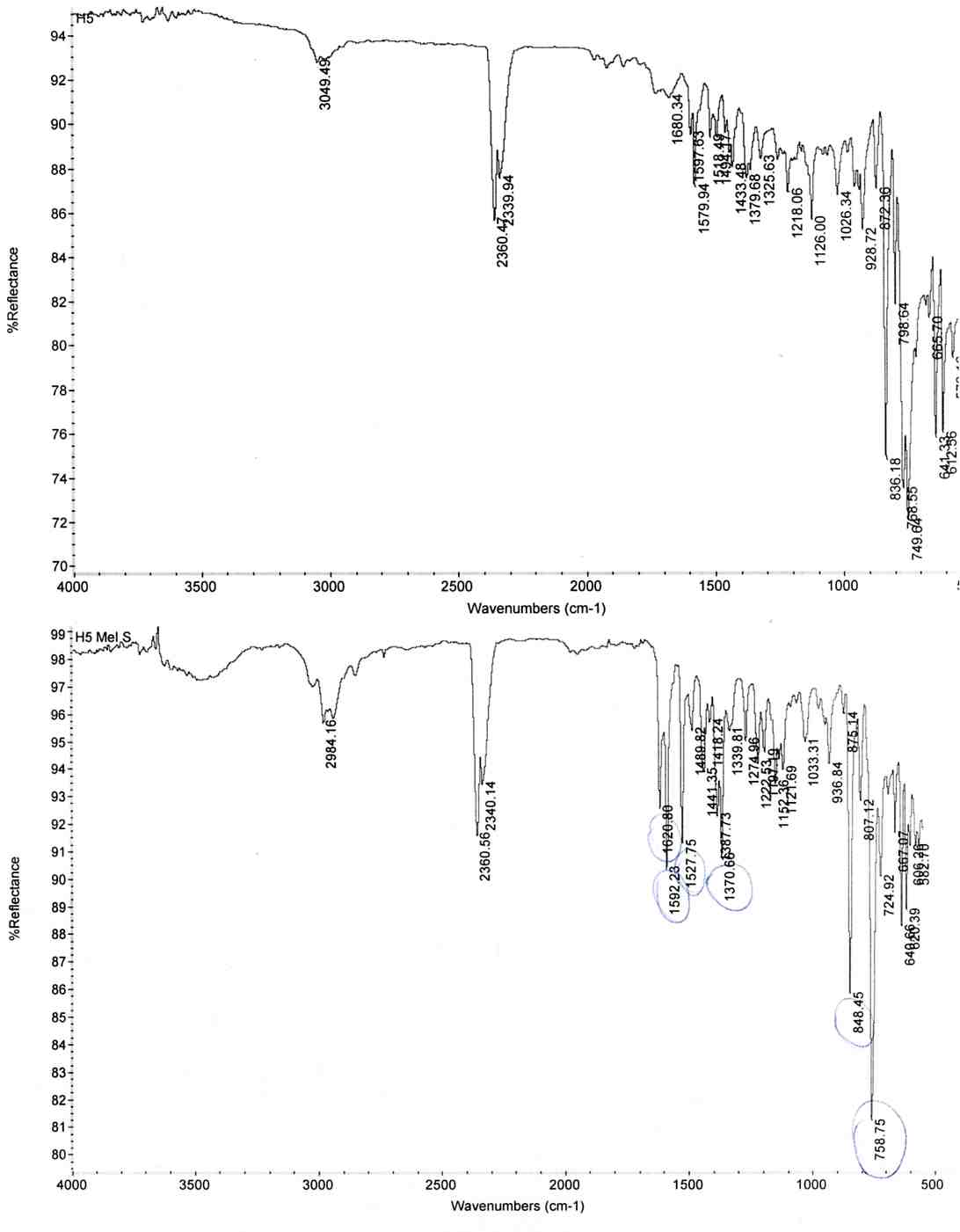


UV-Vis in CH_2_Cl_2_ for (**5a**): nm (log ε) 232 (2.23), 257, (1.89), 319 (1.65), 393 (1.26). Compound **5a** also shows a non-structured fluorescence band centered at about 470 nm.

*1.5. X-ray Crystallographic Study*

X-ray analysis: C_22_H_16_NI, M_r_ = 421.3, yellow crystals , size 0.78 x 0.20 x 0.07 mm, monoclinic, space group P2_1_/n, a= 7.922(1), b = 18.892(3), c = 12.060(1) Å, β = 105.51(1)°, V = 1739.2.(4) Å^3^,
Z = 4, D_c_ = 1.61 g cm^−3^, F(000) = 832; λ = 0.71071 Å, room temperature. Bruker SMART-APEX CCD area detector diffractometer. Total of 27123 reflections (3413 unique, R_int_ = 0.023) were collected up to 52° in 2θ and index range: −9 ≤ h ≥ 9, −23 ≤ k ≥ 23, −14 ≤ l ≥ 14. Empirical adsorption correction was applied using SADABS PROGRAM; the structure was solved by direct methods using SIR97 program and refined on F^2^ by full-matrix least-squares procedure with SHELXL97 for 218 parameters, with anisotropic temperature factors for non-H atoms. The final stage converged to
R = 0.032 (R_w_ = 0.090) for 3152 observed reflections, with I ≥ 2σ(I), and R = 0.035 (R_w_ = 0.092) for all unique reflections after merging. Goof = 0.994, maximum and minimum residual peaks of 1.24 (in proximity of the iodide ion) and −0.20e Å^−3^, respectively.

CCDC 670924 contains the supplementary crystallographic data for this structure. These data can be obtained free of charge from The Cambridge Crystallographic Data Centre via www.ccdc.ac.uk/data_request/cif.

In the crystal, molecules of opposite chirality alternate in chains elongated along *c* direction, while molecules of the same chirality are stacked in antiparallel chains along the *a* direction, as shown in Figure S3.

**Figure S3.** An arbitrary view of the crystal packing of **5a**.


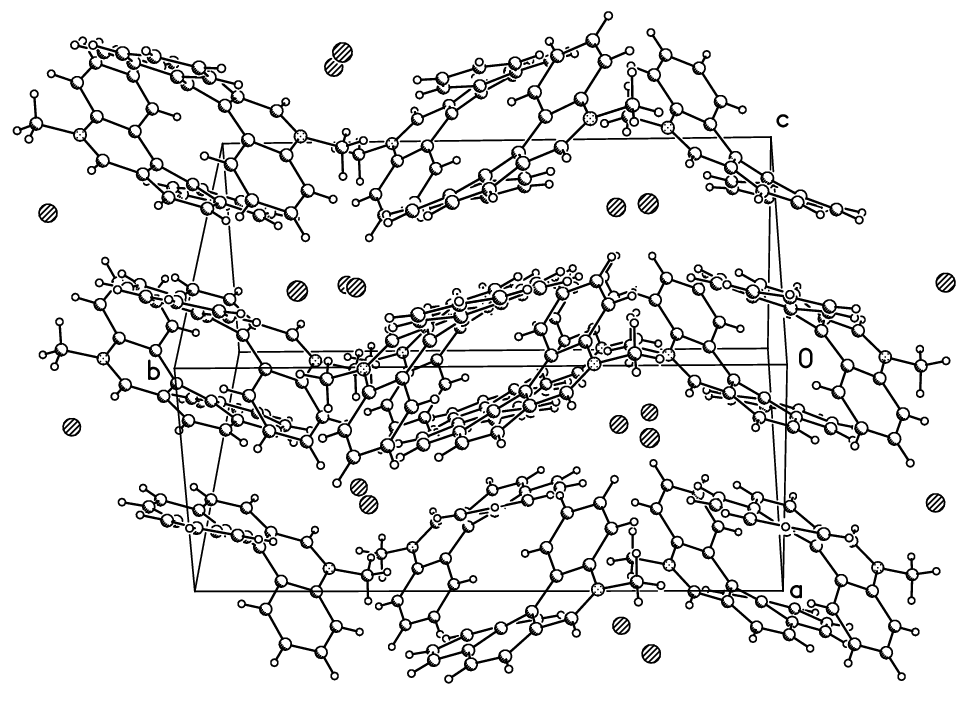


**2.** **Methylation of Diazahelicene 9**

**Figure S4.** ^1^H-NMR spectrum of the mixture of **9a** and **9b**.

**
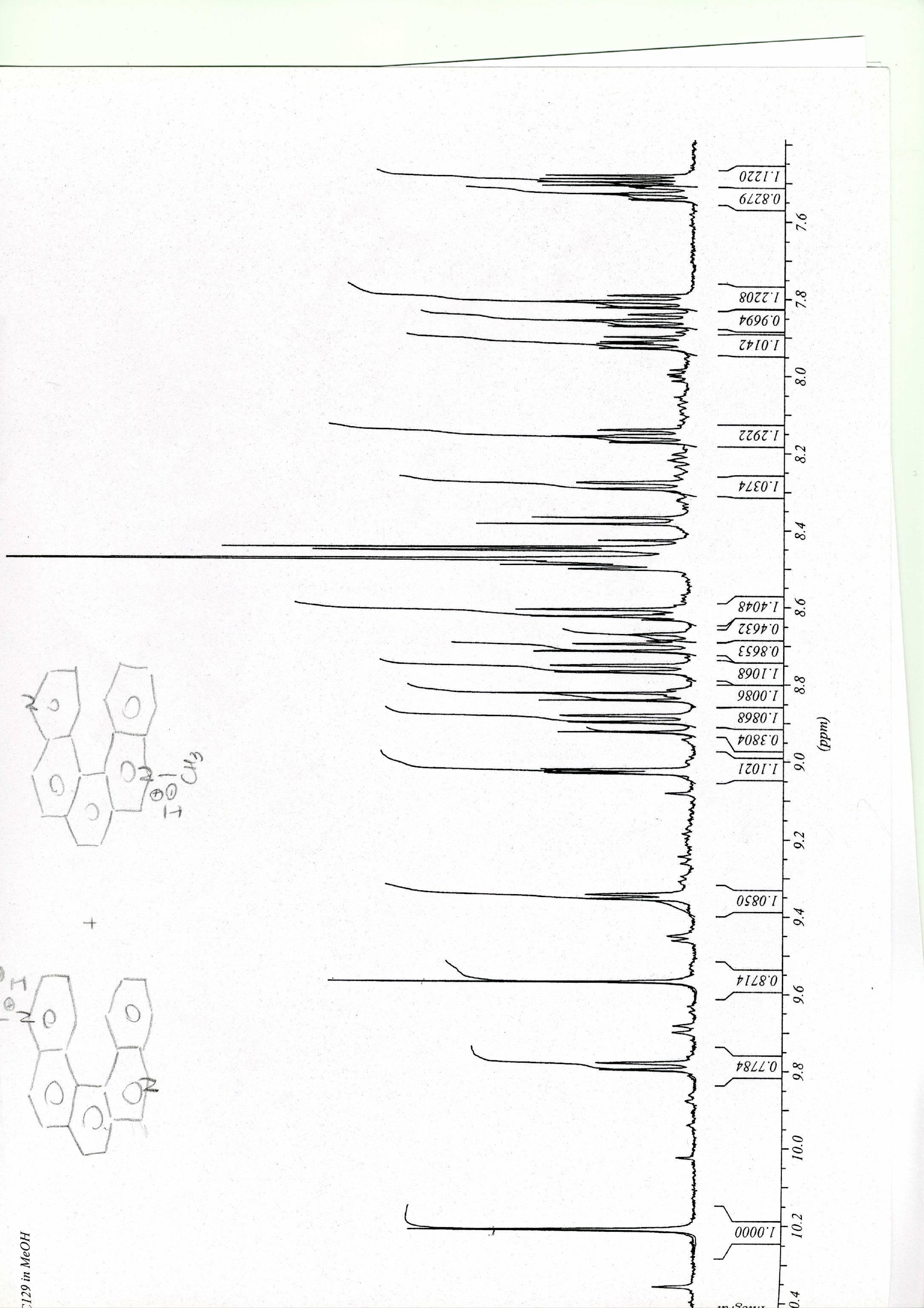
**

**3. Complex 14**

**Figure S5.** Comparison between ^31^P-NMR spectra of precursor [PtCl_2_(NCMe)(PPh_3_)] (lower, blue) and product [PtCl_2_(PPh_3_)(H5)] (upper, red).


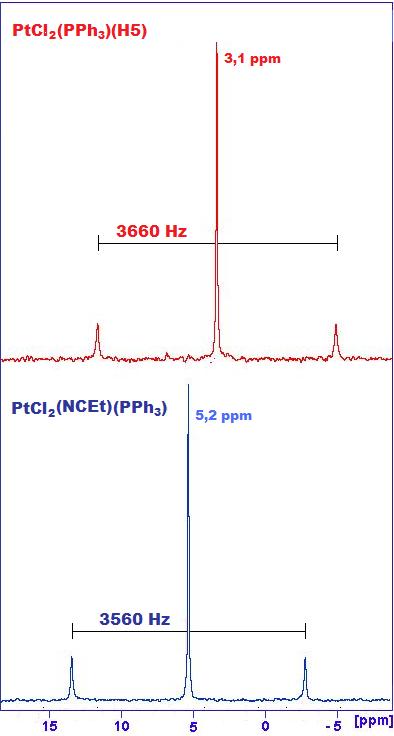


**Figure S6.** Comparison between ^195^Pt NMR spectra of precursor [PtCl_2_(NCMe)(PPh_3_)] (lower, blue) and product [PtCl_2_(PPh_3_)(H5)] (upper, red).


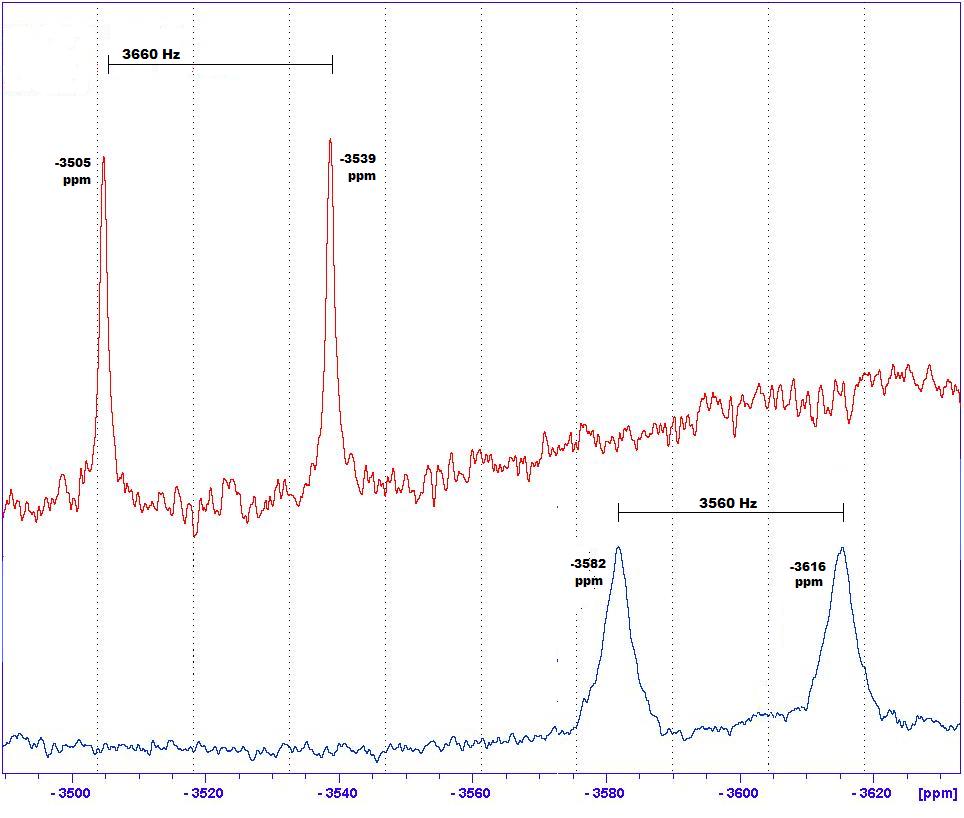

Supplement: Supplementary file 1 [file molecules-17-00463-s001.doc]
